# A computationally designed antigen eliciting broad humoral responses against SARS-CoV-2 and related sarbecoviruses

**DOI:** 10.1038/s41551-023-01094-2

**Published:** 2023-09-25

**Authors:** Sneha Vishwanath, George William Carnell, Matteo Ferrari, Benedikt Asbach, Martina Billmeier, Charlotte George, Maria Suau Sans, Angalee Nadesalingam, Chloe Qingzhou Huang, Minna Paloniemi, Hazel Stewart, Andrew Chan, David Arthur Wells, Patrick Neckermann, David Peterhoff, Sebastian Einhauser, Diego Cantoni, Martin Mayora Neto, Ingo Jordan, Volker Sandig, Paul Tonks, Nigel Temperton, Simon Frost, Katharina Sohr, Maria Teresa Lluesma Ballesteros, Farzad Arbabi, Johannes Geiger, Christian Dohmen, Christian Plank, Rebecca Kinsley, Ralf Wagner, Jonathan Luke Heeney

**Affiliations:** 1 Lab of Viral Zoonotics, Department of Veterinary Medicine, University of Cambridge, Cambridge, UK; 2 DIOSynVax Ltd, University of Cambridge, Cambridge, UK; 3 Institute of Medical Microbiology and Hygiene, University of Regensburg, Regensburg, Germany; 4 Department of Pathology, University of Cambridge, Cambridge, UK; 5 Institute of Clinical Microbiology and Hygiene, University Hospital Regensburg, Regensburg, Germany; 6 Viral Pseudotype Unit, Medway School of Pharmacy, The Universities of Kent and Greenwich at Medway, Chatham, UK; 7 ProBioGenAG, Berlin, Germany; 8 London School of Hygiene and Tropical Medicine, London, UK; 9 Microsoft Health Futures, Redmond, WA USA; 10 Ethris GmbH, Planegg, Germany

**Keywords:** Biological techniques, Biotechnology, Immunology

## Abstract

The threat of spillovers of coronaviruses associated with the severe acute respiratory syndrome (SARS) from animals to humans necessitates vaccines that offer broader protection from sarbecoviruses. By leveraging a viral-genome-informed computational method for selecting immune-optimized and structurally engineered antigens, here we show that a single antigen based on the receptor binding domain of the spike protein of sarbecoviruses elicits broad humoral responses against SARS-CoV-1, SARS-CoV-2, WIV16 and RaTG13 in mice, rabbits and guinea pigs. When administered as a DNA immunogen or by a vector based on a modified vaccinia virus Ankara, the optimized antigen induced vaccine protection from the Delta variant of SARS-CoV-2 in mice genetically engineered to express angiotensin-converting enzyme 2 and primed by a viral-vector vaccine (AZD1222) against SARS-CoV-2. A vaccine formulation incorporating mRNA coding for the optimized antigen further validated its broad immunogenicity. Vaccines that elicit broad immune responses across subgroups of coronaviruses may counteract the threat of zoonotic spillovers of betacoronaviruses.

## Abstract (web summary)

A computationally designed antigen based on the receptor binding domain of the spike protein of sarbecoviruses elicits broad humoral responses against severe acute respiratory syndrome viruses in mice, rabbits and guinea pigs.

## Main

Among the coronaviruses of the greatest pandemic risk are the viruses of the *Betacoronavirus* genus that bind to angiotensin-converting enzyme 2 (ACE-2)^1,2^. Over the past two decades, two ACE-2-binding sarbecoviruses have spilled over into human populations, causing the severe acute respiratory syndrome (SARS) epidemic in 2002–2003 and the current SARS coronavirus 2 (SARS-CoV-2) pandemic. Bats are a reservoir of a large number of SARS-CoV-like ACE-2-binding sarbecoviruses which pose a constant threat for future spillover into humans, with the potential to cause new epidemics^3,4^. In addition to the emergence of new ACE-2-binding viruses from zoonotic reservoirs, another concern is the emergence of mutations in variants of these viruses that are capable of escaping vaccine-induced immunity—a constant observation and concern in the current pandemic. As human infections increase globally during the pandemic, the virus has continued to accrue mutations, most notably in the spike protein^5^. An accumulating number of variants of concern (VOCs) have implications for increased transmission and escape from natural and vaccine-induced immunity^6–9^. The N501Y (asparagine to tyrosine) substitution in the receptor binding domain (RBD) of the spike protein is a common feature of VOCs and is associated with increased affinity of the viral spike protein to the ACE-2 receptor and with a subsequent increase in transmission^10^. Notably, the majority of these mutations reported in VOCs are in the receptor binding motif in the RBD (or around it), which interacts with ACE-2, as well as in the regions that induce highly potent neutralizing antibodies^11,12^. In the key RBD epitopes, the Delta VOC^13^ has L452R and T478K mutations, whereas the Omicron lineage VOCs have multiple mutations^14^. The continued emergence of these VOCs during the ongoing coronavirus disease 2019 (COVID-19) pandemic and the constant threat of new zoonotic spillovers of coronaviruses from animals to humans highlight the need for next generation vaccines with broader protection from ACE-2-binding sarbecoviruses as well as from the emerging VOCs.

In this study, to increase the coverage to all the viruses of the *Sarbecovirus* subgenus of betacoronaviruses, we used a digitally immune-optimized synthetic vaccine (DIOSynVax) technology to design antigens. These computationally immune-optimized and structurally engineered antigens are selected in vivo to induce immune responses across a group of related viruses. First, we generated a phylogenetically informed RBD-based antigen by comparing all the known human and animal reservoir *Sarbecovirus* sequences. This antigen design was further used as a backbone for designing both epitope-optimized and immune-refocused designs using available structural data for the spike protein in complex with RBD-binding monoclonal antibodies, in this case specifically those that bound both SARS-CoV and SARS-CoV-2, such as S309 (ref. ^15^) and CR3022 (ref. ^16^). The nucleic acid sequences of these in-silico-designed antigens were optimized for expression in human cells, and synthetic genes expressing each unique antigen were shuttled in an expression cassette for consecutive in vitro and in vivo screens in Bagg albino laboratory (BALB/c) mice. The best-in-class immunologically optimal antigen, which we designated as ‘T2_17’, was further validated by DNA immunization screens in guinea pigs and rabbits. To further validate the utility of this antigen to boost specific responses on the background of spike-specific immune responses to pre-existing early Wuhan isolates (used by most licensed vaccines), the T2_17 antigen was administered as a heterologous boost using either DNA or MVA immunogens to transgenic mice expressing human ACE-2 (K18-hACE-2) previously primed with the AZD1222 vaccine for SARS-CoV-2. RBD-specific immune responses were observed in groups immunized with the T2_17 antigen. Further immunogenicity of the T2_17 antigen was confirmed in mice and guinea pigs as a messenger (m)RNA-delivered immunogen based on chemically modified mRNA^17^ in a lipidoid nanoparticle formulation (LNP)^18^. These studies confirmed that these computationally derived antigens can induce broad humoral responses, using a single RBD-based antigen covering SARS-CoV, SARS-CoV-2 (including VOCs) and related bat sarbecoviruses.

## Results

### In silico design of antigens

**Fig. 1:**
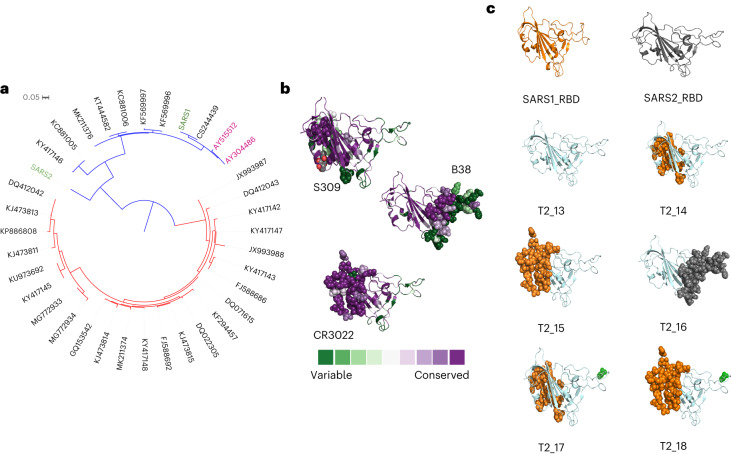
In silico design of antigen candidates. **a**, Phylogenetic tree generated for sarbecoviruses using the protein sequence of the RBD of the spike proteins. The tree was generated using IQ-TREE^37^. Human viruses are represented in green, palm civet viruses in pink and bat viruses in dark grey. The two distinct clades are coloured in red (non-ACE-2 binding) and blue (ACE-2 binding). **b**, Structural models of RBD with epitope regions highlighted as spheres. The backbone of RBD is coloured according to the conservation of amino acid as calculated by the CONSURF algorithm^59^ using the alignment utilized for construction of the phylogenetic tree. The figure was generated and rendered using PyMOL^60^ utilizing PDB^39^ IDs 6WPS (ref. ^15^), 6W41 (ref. ^16^) and 7BZ5 (ref. ^11^). **c**, Structural representation of the different antigen designs used in the study. The epitopes that were modified to match the wild-type SARS-CoV (coloured orange) and wild-type SARS-CoV-2 (coloured grey) are represented as spheres. The further glycosylation site modification is represented in green.

Sequences of spike protein of viruses belonging to the *Sarbecovirus* subgenus were compiled from the National Centre for Biotechnology Information (NCBI) virus database^19^ and further pruned to remove poor-quality and redundant sequences. The hCoV-19/Wuhan/IVDC-HB-01/2019 strain of SARS-CoV-2 was used for the analyses. The phylogenetic tree of these sequences is presented in Fig. 1a. Two distinct clades are observed in the tree: clade 1 viruses, which do not interact with the ACE-2 receptor^1,20^, and clade 2 viruses, which do. Clade 1 viruses share many of the sequence features of the members of clade 2 but possess deletions within the ACE-2 binding region (Supplementary Fig. 1). An optimized core sequence (T2_13) was designed, such that the antigen was phylogenetically the closest to all the sarbecoviruses represented in the phylogenetic tree shown in Fig. 1a. Due to the evolutionary relatedness between the input sequences and the evolution model used in the pipeline, the design captures both the conserved as well as distinct epitopes of the input sequences. To further understand the importance of the amino acid composition of epitopes in generating antibody responses, we modified T2_13 to display the exact amino acid sequences of epitopes of SARS-CoV for monoclonal antibodies S309 (ref. ^15^) (T2_14) and CR3022 (ref. ^16^) (T2_15) and of SARS-CoV-2 for monoclonal antibody B38 (ref. ^11^) (T2_16). The sequences of epitopes for monoclonal antibodies S309 (ref. ^15^) and CR3022 (ref. ^16^) are highly conserved across the sequences considered in this study, while the sequence of epitopes for monoclonal antibody B38 (ref. ^11^) is highly divergent (Fig. 1b). These antibodies were chosen for the analyses, as these were the only biochemically and structurally characterized antibodies available at the time of generating the antigen designs. We further modified the epitope region for monoclonal antibody B38 (ref. ^11^) by introducing a glycosylation site on the backbone of T2_14 (T2_17) and T2_15 (T2_18). This was done to mask the divergent epitope region and enhance the presentation of the conserved epitopes to the immune system. The masking of epitopes by introducing glycans has been exploited by many viruses such as hepatitis C virus^21^, Lassa virus^22^ and influenza virus^23^ to escape natural immunity and we used this strategy to train the immune system to elicit preferential immune response towards the conserved but subdominant epitopes. To compare the immunogenicity of soluble and membrane-anchored RBD-based vaccines, membrane-anchored forms of T2_13 and T2_17 (T2_13_TM and T2_17_TM, respectively) were generated. The structural stability of these designs was evaluated in silico using the BUILD module of the FoldX^24^ algorithm, with T2_13 as the reference model. Structural models of these vaccine antigens are presented in Fig. 1c.

### Antigen selection and immunogenicity confirmation in BALB/c mice

**Fig. 2:**
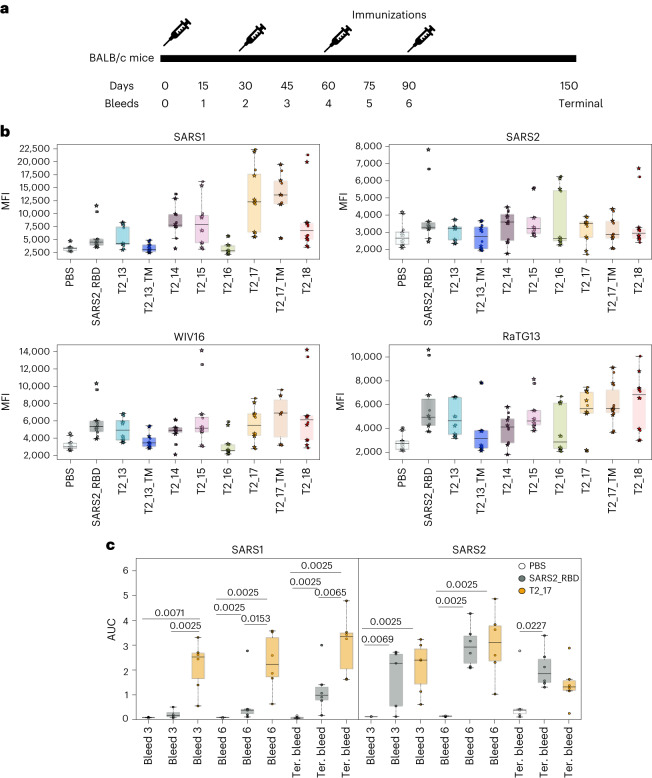
In vitro selection and in vivo immunogenicity of antigens in BALB/c mice. **a**, Immunization and bleed schedule of BALB/c mice. Mice were immunized at intervals of 30 d and bled every 15 d. **b**, FACS binding data for the antigens. Sera from mice immunized with designed antigens were screened for binding to SARS-CoV, SARS-CoV-2, WIV16 and RaTG13 spike proteins. The *x* axis represents all the vaccine designs considered for screening, and the *y* axis represents the mean fluorescence intensity (MFI). For each mouse serum, two replicates of MFI (represented as filled circles and filled stars) are reported. **c**, Elicitation of binding antibodies against SARS-CoV and SARS-CoV-2 by T2_17 was confirmed using ELISA, with SARS-CoV-2 RBD as control vaccine design. T2_17 generated cross-binding antibodies. The *x* axis represents the bleeds, and the *y* axis represents the area under the curve (AUC) from ELISA binding curves. The boxes represent the quartiles (25th, 50th and 75th percentiles) of the distribution, the whiskers represent the minimum and maximum (excluding outliers) and the fliers represented as filled circles denote outliers. Two-tailed Mann–Whitney *U* tests demonstrated statistical significance. *n* = 6 for **b** and **c**.

In vivo screening in BALB/c mice was performed by immunizing mice with the in silico designed antigens and SARS-CoV-2 RBD (hCoV-19/Wuhan/IVDC-HB-01/2019) as a DNA immunogen (Fig. 2a). The sera from immunized mice were assessed for cross-reactive antibodies against spike proteins in a flow cytometry-based cell-surface display assay. Binding against four spike proteins, namely SARS-CoV (SARS-Tor2), SARS-CoV-2 (hCoV-19/Wuhan/IVDC-HB-01/2019), WIV16 and RaTG13, were tested. Sera taken 2 weeks following the second immunization with the antigen designs demonstrated the binding profile of sera induced by the vaccine candidates for different spike proteins (Fig. 2b). Sera from all antigen-immunized mice showed higher binding than those of PBS-immunized mice across the four spike proteins, suggesting seroconversion of the mice on immunization with the antigens.

Across the four spike proteins, no significant differences in binding were observed for sera from mice immunized with T2_13 and sera from mice immunized with SARS-CoV-2 RBD (all *P* > 0.05, Mann–Whitney *U* (MWU) test), suggesting that epitopes in this design are biased towards SARS-CoV-2 RBD. For the T2_16 design, in which the epitope region for mAb B38 was mutated to the epitope region on SARS-CoV-2, binding to SARS-CoV, WIV16 and RaTG13 declined in comparison with T2_13 (*P* < 0.05, MWU test) without significant changes in binding to SARS-CoV-2. Matching of the epitopes of S309 and CR3022 to SARS-CoV (T2_14 and T2_15) enhanced the binding to SARS-CoV spike (*P* < 0.05, MWU test) but not to other spike proteins. Introduction of a glycosylation site in design T2_17 significantly enhanced the binding of elicited antibodies to SARS-CoV and RaTG13 (*P* < 0.01, MWU test) in comparison with T2_14, but no difference was observed in T2_18 in comparison with T2_15. There was no statistical difference between transmembrane-anchored and soluble designs when delivered as a DNA immunogen. As T2_17 has either the best (or second best) median binding to the four spike proteins, we choose T2_17 as the lead candidate for further immunological assays.

Elicitation of cross-binding antibodies by T2_17 was further confirmed by enzyme-linked immunosorbent assay (ELISA) with SARS-CoV RBD and SARS-CoV-2 RBD (Fig. 2c), revealing robust binding antibody responses to both SARS-CoV and SARS-CoV-2 within 2 weeks of the second immunization. T2_17 elicited stronger responses against SARS-CoV in comparison with SARS-CoV-2 RBD. Against SARS-CoV-2, the two antigens SARS-CoV-2 RBD and T2_17 generated similar binding antibody responses.

### Immunogenicity confirmation of T2_17 in outbred animals

**Fig. 3:**
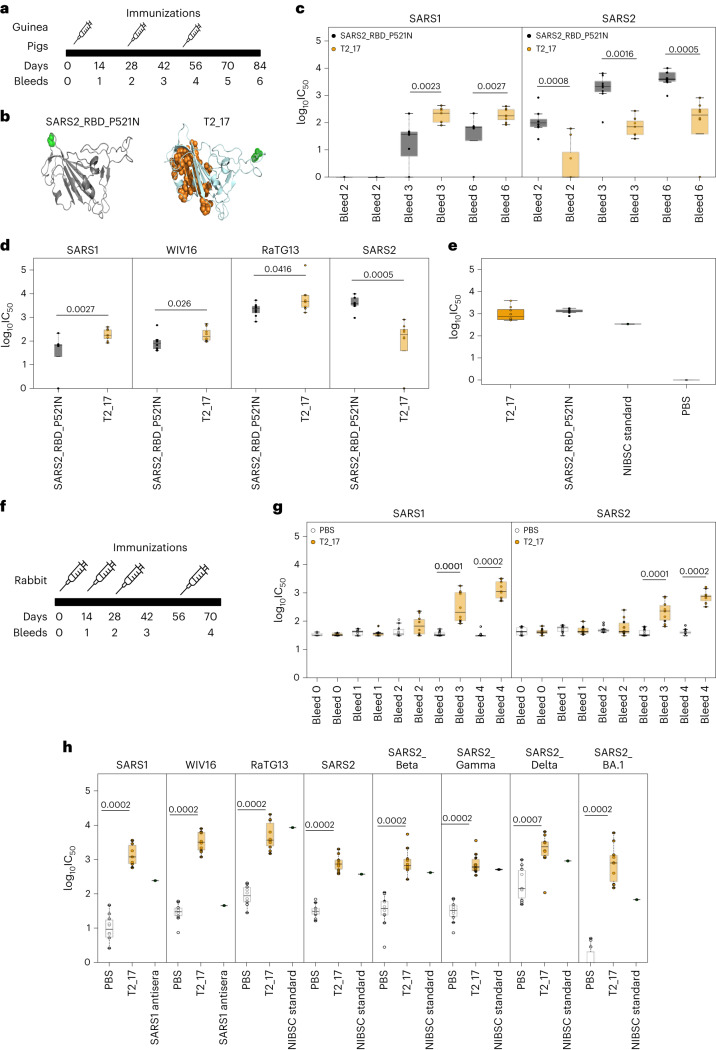
Immunogenicity studies in guinea pigs and rabbits. **a**, Immunization and bleed schedule of guinea pigs. Guinea pigs were immunized with DNA delivered intradermally using the Tropis PharmaJet device at 28-d intervals and bled every 14 d. **b**, Structure models of the antigen designs used for the study in guinea pigs. The glycosylation site and the modified epitope are represented as green and orange spheres, respectively. **c**, Neutralization of SARS-CoV and SARS-CoV-2 pseudoviruses by guinea pig sera immunized with T2_17 and SARS2_RBD_P521N. The *x* axis represents the bleed number, and the *y* axis represents the log_10_IC_50_ values for neutralization curves. **d**, Broad neutralization of SARS-CoV, WIV16, RaTG13 and SARS-CoV-2 pseudoviruses by T2_17 in comparison with SARS2_RBD_P521N. Sera post 28 d after three immunizations (bleed 6) were used for comparison. **e**, ACE-2 competition ELISA. Sera from guinea pigs immunized with T2_17 and SARS2_RBD_P521N. The NIBSC standard (20/162) was used as control. **f**, Immunization and bleed schedule of rabbits. Rabbits were immunized at intervals of 14 d and bled every 14 d. **g**, Neutralization of SARS-CoV and SARS-CoV-2 pseudoviruses by rabbit sera immunized with T2_17. The *x* axis represents the bleed number, and the *y* axis represents the log_10_IC_50_ values for neutralization curves. **h**, Broad neutralization of SARS-CoV, WIV16, RaTG13, SARS-CoV-2, SARS-CoV-2 Beta, SARS-CoV-2 Gamma, SARS-CoV-2 Delta and SARS-CoV-2 BA.1 pseudoviruses by T2_17. Sera taken 14 d after the fourth immunization (bleed 4) were used for comparison. NIBSC standards for SARS-CoV-2 and SARS-CoV antiserum were used as reference. The boxes represent the quartiles (25th, 50th and 75th percentiles) of the distribution, the whiskers represent the minimum and maximum (excluding outliers) and the fliers represented as filled circles denote outliers. Two-tailed Mann–Whitney *U* tests demonstrated statistical significance. *n* = 8 for **c**, **d** and **e**; 10 for **g** and **h**.

**Extended Data Fig. 1:**
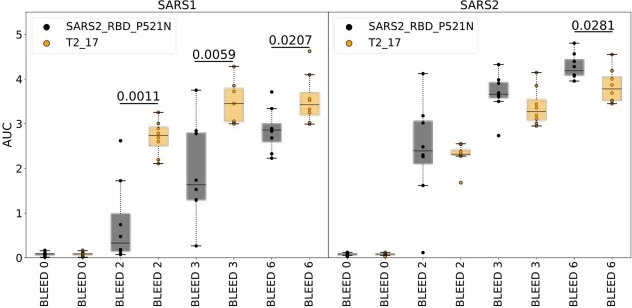
Binding antibody data for T2_17 in guinea pigs. Elicitation of binding antibodies against SARS-CoV and SARS-CoV-2 by T2_17 and SARS2_RBD_P521N was confirmed using ELISA. The pre-bleed (Bleed 0) is considered as the control for non-specific binding. The X-axis represents the bleed number, and the Y-axis represents the area under the curve (AUC) from the ELISA binding curve. The boxes represent the quartiles (25th, 50th and 75th percentiles) of the distribution, and the whiskers represent the minimum and maximum of the distribution (excluding outliers) and the fliers represented as filled circle represent the outliers. Two-tailed Mann-Whitney U demonstrated statistical significance. Sample size is eight.

**Extended Data Fig. 2:**
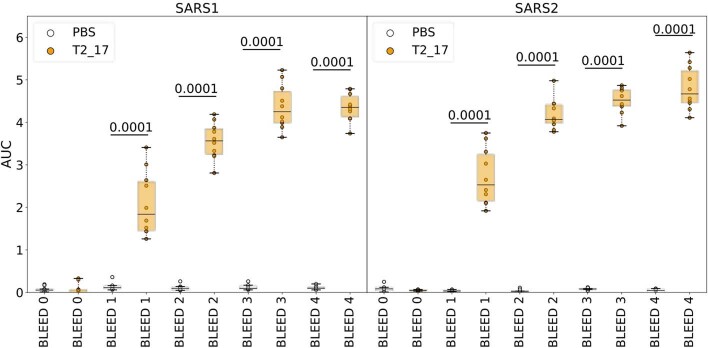
Binding antibody data for T2_17 in rabbits. Elicitation of binding antibodies against SARS-CoV and SARS-CoV-2 by T2_17 was confirmed using ELISA. The X-axis represents the bleed number, and the Y-axis represents the area under the curve (AUC) from the ELISA binding curve. The boxes represent the quartiles (25th, 50th and 75th percentiles) of the distribution, and the whiskers represent the minimum and maximum of the distribution (excluding outliers) and the fliers represented as filled circle represent the outliers. Two-tailed Mann-Whitney U demonstrated statistical significance. Sample size is ten.

To determine the breadth of antibody response and neutralization potency of T2_17 as a DNA immunogen in outbred animals, guinea pigs were immunized using the European conformity approved and clinically validated Pharmajet Tropis needleless, intradermal delivery device to ensure standardized intradermal delivery (Fig. 3a). As a control, we used a C-terminal glycosylation modified SARS-CoV-2 RBD (SARS2_RBD_P521N) (Fig. 3b) which we had previously evaluated in BALB/c mice^25^ (Supplementary Fig. 2). Generation of neutralizing antibodies to both SARS-CoV and SARS-CoV-2 was confirmed using pseudoviruses expressing full-length spike proteins of SARS-CoV and SARS-CoV-2. While both T2_17 and SARS2_RBD_P521N generated binding antibodies against both SARS-CoV and SARS-CoV-2 (Extended Data Fig. 1) after one immunization, T2_17 elicited significantly higher antibodies than SARS2_RBD_P521N to SARS-CoV and comparable antibodies against SARS-CoV-2. Higher titres of binding antibodies were detected for T2_17 to SARS-CoV in comparison with SARS2_RBD_P521N after two immunizations, while the responses were comparable for SARS-CoV-2. After three immunizations, SARS2_RBD_P521N induced a higher response to SARS-CoV-2, while T2_17 had higher responses to SARS-CoV (Extended Data Fig. 1). Neutralizing antibodies were detected for SARS-CoV-2 after the first immunization, while significant neutralizing responses to SARS-CoV developed after two immunizations, although these were more potent from T2_17 than from SARS2_RBD_P521N (Fig. 3c). Higher binding and neutralizing responses by SARS2_RBD_P521N to SARS-CoV-2 were expected as it differs from SARS-CoV-2 by only one amino acid. To further confirm whether the T2_17 antigen generates broader responses than SARS2_RBD_P521N, we compared sera induced by these two antigens 28 d post third immunization for neutralization against SARS-CoV (SARS-Tor2), SARS-CoV-2 (hCoV-19/Wuhan/IVDC-HB-01/2019), WIV16 and RaTG13 pseudoviruses. Statistically significant higher neutralizing antibody titres were generated by T2_17 against SARS-CoV, WIV16 and RaTG13 (Fig. 3d). To further confirm that anti-sera to T2_17 could abrogate hACE-2 receptor binding, we performed an ELISA-based competition assay (Fig. 3e), demonstrating that T2_17 and SARS2_RBD_P521N anti-sera abrogated binding to the hACE-2 receptor and are comparable to the WHO standard of pooled convalescent COVID-19 patient sera (NIBSC standard 20/162). These findings demonstrated important proof-of-concept of T2_17 as a single gene-delivered, structurally engineered antigen capable of eliciting broad pan-sarbecovirus neutralizing antibodies. Before clinical trials in humans, a good manufacturing practice lot of T2_17 DNA was manufactured and evaluated for safety and immunogenicity in rabbits using the same gene delivery device to ensure uniform intradermal administration (Fig. 3f). After one immunization, binding antibodies to SARS-CoV and SARS-CoV-2 were elicited (Extended Data Fig. 2), increasing on subsequent immunizations until a plateau was reached by the fourth immunization. Robust neutralizing antibodies were observed 2 weeks following the third immunization (Fig. 3g). Sera post 14 d after four immunizations (bleed 4) showed broad neutralizing antibody responses against the SARS-CoV, SARS-CoV-2, Beta, Gamma, Delta and Omicron (BA.1) VOCs and bat sarbecoviruses, WIV16 and RaTG13 (Fig. 3h).

### Challenge studies in mice expressing K18-hACE-2

**Fig. 4:**
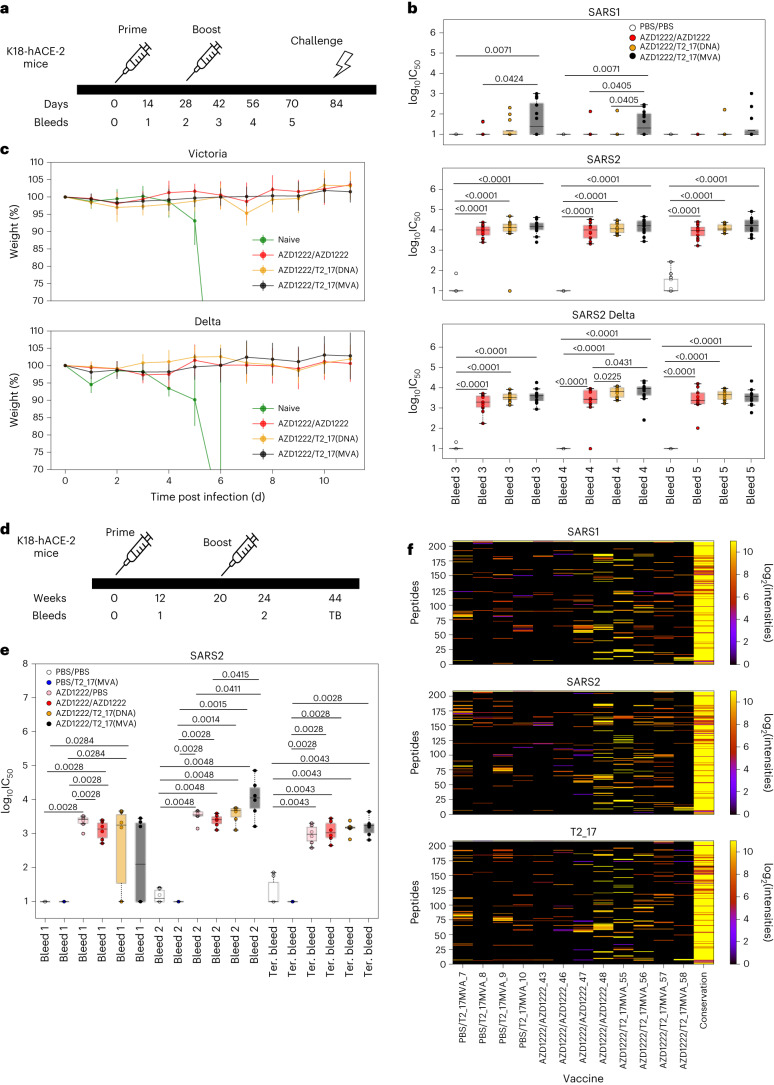
Immunogenicity and challenge studies in K18-hACE-2 mice. **a**, Immunization, bleed and challenge schedule of K18-hACE-2 mice. K18-hACE-2 mice were primed with AZD1222 vaccine and then boosted with either AZD122 or T2_17 after 4 weeks. The mice were challenged after 8 weeks with either the Victoria strain of SARS-CoV-2 or the Delta variant. **b**, Neutralization of SARS-CoV, SARS-CoV-2 and the Delta variant of SARS-CoV-2 pseudoviruses by K18-hACE-2 mice sera. Sera of mice boosted with T2_17(DNA) and T2_17(MVA) significantly neutralized the Delta variant (B.1.617.2) in comparison with those boosted by AZD1222 at bleed 4. The *x* axis represents the bleed number, and the *y* axis represents the log_10_IC_50_ values for neutralization curves. **c**, Weight loss profile of K18-hACE-2 mice following challenge with the Victoria strain and the Delta variant. All mice except the naive were protected. **d**, Immunization and bleed schedule of K18-hACE-2 mice for longitudinal analysis. **e**, Neutralization of SARS-CoV-2 pseudoviruses by K18-hACE-2 mice sera. Neutralization by sera of mice boosted with T2_17(MVA) is statistically higher than that by sera of mice boosted with AZD1222 at bleed 2. The *x* axis represents the bleed number, and the *y* axis represents the log_10_IC_50_ values for neutralization curves. **f**, Peptide microarray analyses of the ACE-2 longitudinal analysis. The *x* axis represents the mice sera, and the *y* axis represents the different linear peptides. The last column represents the conservation of the corresponding peptide in SARS-CoV, SARS-CoV-2 and T2_17. The boxes represent the quartiles (25th, 50th and 75th percentiles) of the distribution, the whiskers represent the minimum and maximum (excluding outliers) and the fliers represented as filled circles denote outliers. Two-tailed Mann–Whitney *U* tests demonstrated statistical significance. *n* = 12 for **b**; 6 for **c** and **e**.

As almost all the human population is seroconverted either due to natural infection, vaccination or both, we tested the efficacy of the T2_17 antigen when given as a booster following AZD1222 (ChAdOx1 nCoV-19) as prime vaccine. Although the present immune landscape of the human population is complex due to multiple immunizations and infections by different SARS-CoV-2 variants, we aimed to study the baseline immunogenicity of T2_17 in the background of vaccination. To address this, homozygous K18-hACE-2 transgenic mice were immunized with 1.4 × 10^9^ viral particles of AZD1222 and 4 weeks later boosted with either T2_17 or the licensed AZD1222 vaccine (Fig. 4a), while the control group received only PBS with each immunization. We administered T2_17 either as a DNA immunogen or a modified vaccinia virus Ankara (MVA) immunogen. The ChadOx-MVA prime–boost regime has been shown to be effective in Ebola^26,27^. Eight weeks post boost, all groups of mice were challenged with either a January 2020 isolate of SARS-CoV-2 (Victoria) or the Delta variant of SARS-CoV-2 (Table 1).

**Table 1:** Prime–boost regime of the K18-hACE-2-mice challenge

Prime	Boost	Challenge
PBS	PBS	Victoria
AZD1222	AZD1222	Victoria
AZD1222	T2_17(DNA)	Victoria
AZD1222	T2_17(MVA)	Victoria
PBS	PBS	Delta
AZD1222	AZD1222	Delta
AZD1222	T2_17(DNA)	Delta
AZD1222	T2_17(MVA)	Delta

**Extended Data Fig. 3:**
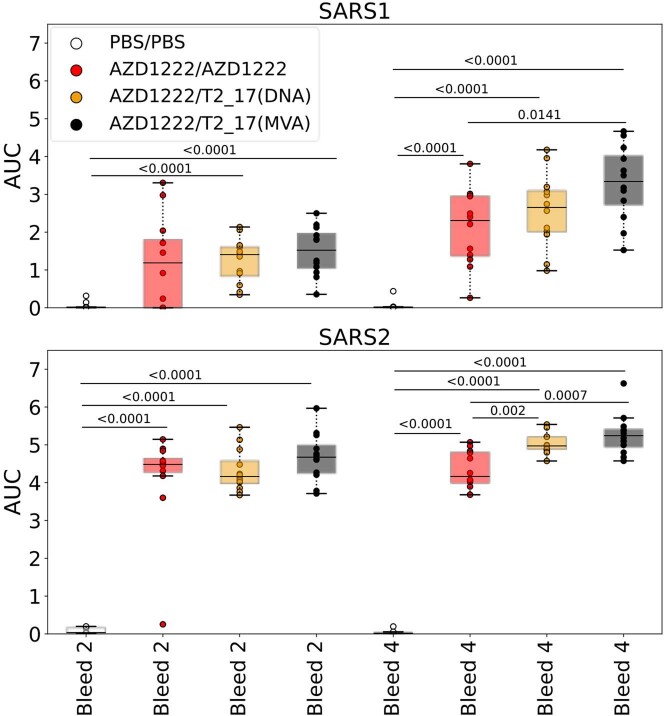
ELISA binding data of K18-hACE2 sera. Binding antibodies were observed 4 weeks post immunization with AZD1222 and 4 weeks post boosting. The X-axis represents the bleed number, and the Y-axis represents the area under the curve (AUC) from ELISA binding curves. The boxes represent the quartiles (25th, 50th and 75th percentiles) of the distribution, and the whiskers represent the minimum and maximum of the distribution (excluding outliers) and the fliers represented as filled circle represent the outliers. The boxes represent the quartiles (25th, 50th and 75th percentiles) of the distribution, and the whiskers represent the minimum and maximum of the distribution (excluding outliers) and the fliers represented as filled circle represent the outliers. Two-tailed Mann-Whitney U demonstrated statistical significance, Sample size is twelve.

Increased binding antibody titres to both SARS-CoV and SARS-CoV-2 after boosting with either AZD1222 or T2_17 were observed (Extended Data Fig. 3). Statistically significant differences in antibody titres to SARS-CoV-2 were observed 4 weeks after boosting with T2_17 as DNA or MVA immunogen in comparison with boosting with AZD1222, while a statistically significant increase in binding antibody titres to SARS-CoV was observed on boosting with T2_17 as MVA immunogen (Extended Data Fig. 3). Generation of neutralizing antibodies to SARS-CoV, SARS-CoV-2 and the Delta VOC was confirmed using pseudoviruses expressing full-length spike proteins of SARS-CoV, SARS-CoV-2 and the Delta VOC. Neutralizing antibodies for SARS-CoV-2 and the Delta VOC were detected for all of the groups, except the control group before challenge, while neutralizing antibodies against SARS-CoV were detected only for the T2_17 MVA-boosted group (Fig. 4b). Two weeks post boost, the antibodies elicited by T2_17 both as DNA and MVA immunogens neutralized the Delta variant significantly better than the sera from mice boosted with AZD1222 (Fig. 4b). Mice from all the groups, except the controls, survived and continued to gain weight following challenge with either the Victoria strain or the Delta variant (Fig. 4c).

### Longitudinal serology study in K18-hACE-2 mice

As all the groups were protected in the challenge studies and similar levels of neutralizing antibodies were observed for SARS-CoV-2, we explored whether this could be due to the short interval between the prime and the boost in the challenge study. For this, we primed another group of K18-hACE-2 mice with AZD1222 vaccine and boosted the mice 20 weeks afterwards (Fig. 4d). The mice were boosted with either AZD1222, T2_17(DNA), T2_17(MVA) or PBS (Table 2). A group of PBS primed mice was immunized with T2_17(MVA) at week 20 as a control. The AZD1222/PBS group was included to monitor the antibody titres over time in the absence of the boost.

**Table 2:** Prime–boost regime of the longitudinal serology study in K18-hACE-2 mice

Prime	Boost
PBS	PBS
PBS	T2_17(MVA)
AZD1222	PBS
AZD1222	AZD1222
AZD1222	T2_17(DNA)
AZD1222	T2_17(MVA)

Only neutralizing antibody titres against SARS-CoV-2 were measured for this longitudinal analysis. In this study as well, significantly higher titres were again observed for the T2_17(MVA)-boosted group (Fig. 4e) at 4 weeks post boost. No antibody titres were observed in the T2_17(MVA) primed group, suggesting that MVA is a weaker immunogen when delivered only as prime. The antibody levels were maintained up to 44 weeks post prime. As T2_17 is an RBD-based antigen, we further explored whether higher RBD-specific antibodies were generated on boosting with T2_17 in comparison with boosting with AZD1222. Terminal bleed sera from 4 mice with the highest neutralizing antibody titres for the vaccine groups PBS/PBS, PBS/T2_17(MVA), AZD1222/AZD1222 and AZD1222/T2_17(MVA) were tested against 15-mer peptides with overlap of 14 from SARS-CoV RBD, SARS-CoV-2 RBD and T2_17 using PEPperPRINT microarray technology. The intensities of the vaccination groups were normalized to that of the PBS/PBS mice group. The microarray data are shown in Fig. 4f. A higher number of peptide hits was observed for the T2_17(MVA)-boosted group in comparison with the AZD1222-boosted group, suggesting that the T2_17-boosted group induced a greater number of RBD-specific antibodies.

### Immunogenicity of the vaccine candidate as mRNA

**Fig. 5:**
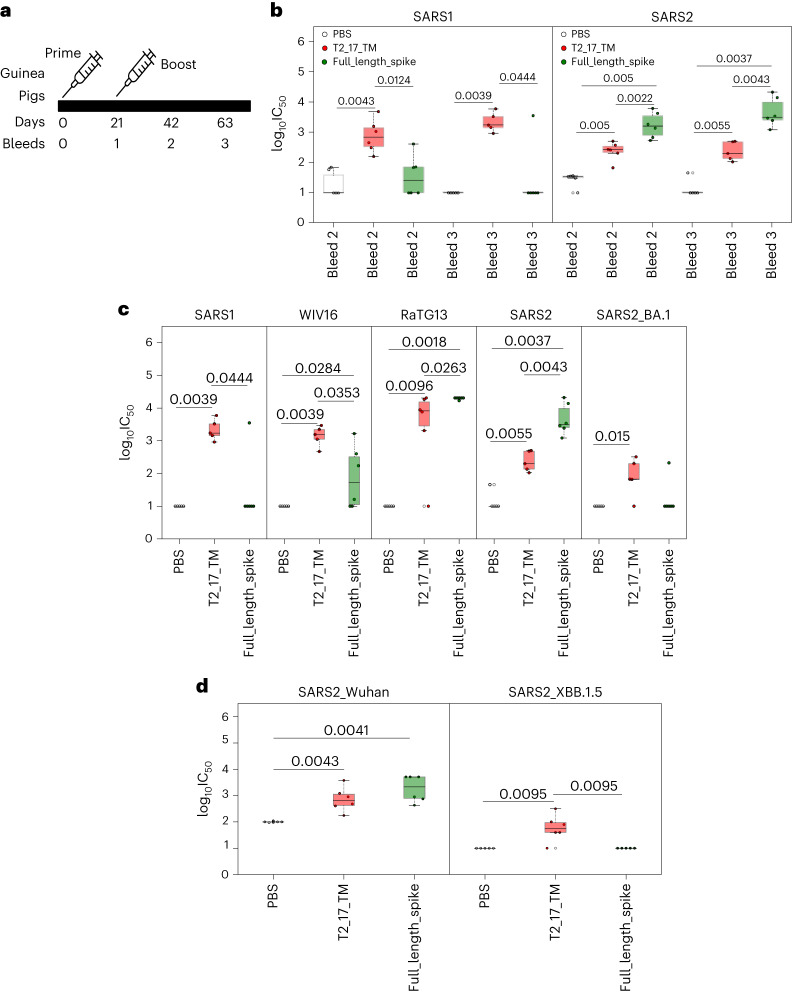
Immunogenicity of mRNA in guinea pigs. **a**, Immunization and bleed schedule of guinea pigs. The guinea pigs were immunized with mRNA at 3-week intervals. **b**, Neutralization of SARS-CoV and SARS-CoV-2 pseudoviruses by guinea pig sera. The *x* axis represents the bleed number, and the *y* axis represents the log_10_IC_50_ values for neutralization curves. **c**, Broad neutralization of SARS-CoV, WIV16, RaTG13, SARS-CoV-2 and SARS-CoV-2 Omicron by T2_17 at a dose of 3.15 µg. Sera taken 6 weeks post boost (bleed 3) were used for comparison. **d**, Neutralization of SARS-CoV-2 and SARS-CoV-2 XBB.1.5 by T2_17 at a dose of 15 µg. Sera taken 6 weeks post boost (bleed 3) were used for comparison. The boxes represent the quartiles (25th, 50th and 75th percentiles) of the distribution, the whiskers represent the minimum and maximum (excluding outliers) and the fliers represented as filled circles denote outliers. Two-tailed Mann–Whitney *U* tests demonstrated statistical significance. *n* = 6 for **b**, **c** and **d**.

**Extended Data Fig. 4:**
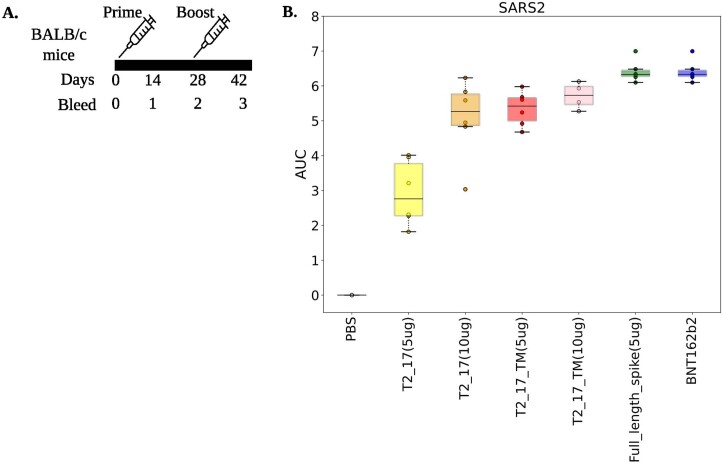
Immunogenicity of mRNA vaccine in BALB/c mice. **(a)** Immunization and bleed schedule of BALB/c mice. The mice were immunized with mRNA at 4-week intervals. **(b)** Elicitation of binding antibodies against SARS-CoV-2 was confirmed using ELISA for sera 2 weeks post boost (bleed 3). The X-axis represents the antigens, and the Y-axis represents the area under the curve (AUC) from ELISA binding curves. The boxes represent the quartiles (25th, 50th and 75th percentiles) of the distribution, and the whiskers represent the minimum and maximum of the distribution (excluding outliers) and the fliers represented as filled circle represent the outliers. Sample size is six.

**Extended Data Fig. 5:**
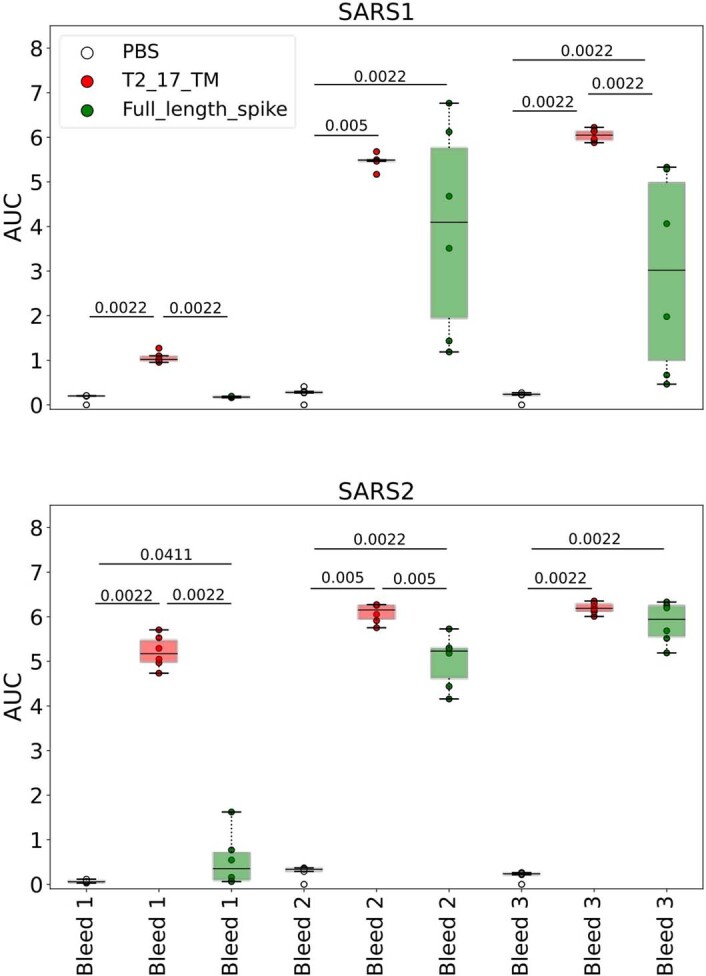
Immunogenicity of mRNA vaccine in guinea pigs. Elicitation of binding antibodies against SARS-CoV and SARS-CoV-2 in guinea pigs was confirmed using ELISA. The X-axis represents the bleeds, and the Y-axis represents the area under the curve (AUC) from ELISA binding curves. The boxes represent the quartiles (25th, 50th and 75th percentiles) of the distribution, and the whiskers represent the minimum and maximum of the distribution (excluding outliers) and the fliers represented as filled circle represent the outliers. Two-tailed Mann-Whitney U demonstrated statistical significance. Sample size is six.

To further validate the immunogenicity of the T2_17 antigen in the mRNA technology, we immunized BALB/c mice with T2_17 as an mRNA immunogen. A previous study on Middle East Respiratory Syndrome (MERS)-based vaccine has shown that membrane-anchored, prefusion-stabilized, full-length MERS spike antigen elicited more potent pseudovirus-neutralizing antibody responses than the soluble form, as mRNA immunogen^28^. To test this, T2_17 was also delivered as a membrane-anchored form (T2_17_TM) mRNA immunogen to mice. The mRNA immunogen (T2_17 and T2_17_TM) was delivered in a prime–boost regime at 4-week intervals in BALB/c mice (Extended Data Fig. 4a) at different doses (5 µg and 10 µg). Full-length spike protein with a double proline mutation in the lipid formulation, similar to the one used for T2_17 and T2_17_TM, was used as a control. In addition, BNT162b2 vaccine was used as a further control. All the antigen-immunized mice generated binding antibodies against SARS-CoV-2. The transmembrane-anchored T2_17 (T2_17_TM) generated higher binding antibody titres at the 5-µg dose in comparison with soluble T2_17 (Extended Data Fig. 4b). No significant difference was observed for T2_17_TM at the two test doses. T2_17 at the higher dose of 10 µg generated binding antibody titres equivalent to those of T2_17_TM (Extended Data Fig. 4b). As higher antibody titres were observed for T2_17_TM at lower doses, we further evaluated immunogenicity of T2_17_TM in guinea pigs. Guinea pigs were immunized either with T2_17_TM at a dose of 3.15 µg or full-length spike with double proline mutations at a dose of 15 µg at 3-week intervals (Fig. 5a). Three weeks post prime, T2_17_TM induced binding antibodies against SARS-CoV as well as SARS-CoV-2, while the full-length spike antigen did not induce binding antibodies against SARS-CoV but induced binding antibodies against SARS-CoV-2 (Extended Data Fig. 5). T2_17_TM induced significantly higher binding antibody titres against SARS-CoV-2 in comparison with full-length spike 3 weeks post boost (Extended Data Fig. 5). Three weeks post boost, higher neutralizing antibody titres against SARS-CoV were observed for T2_17_TM. Three of the guinea pigs immunized with full-length spike induced neutralizing titres against SARS-CoV 3 weeks post boost. Neutralizing titres declined to low levels afterwards in this group but remained high in animals vaccinated with T2_17_TM, although neutralizing antibody titres against SARS-CoV-2 were lower after vaccination with T2_17_TM in comparison with full-length spike. The breadth of antibody immune responses elicited by the T2_17_TM antigen is demonstrated by significantly higher neutralizing titres than those induced by full-length spike against WIV16, SARS-CoV and the SARS-CoV-2 Omicron variant (BA.1) pseudoviruses 6 weeks post boost (Fig. 5c). The neutralizing titres induced by the full-length spike were almost negligible for the SARS-CoV-2 Omicron (BA.1) variant. We further tested for neutralization titre against one of the recent prominent variants, XBB.1.5. No neutralizing titres were observed for both T2_17_TM at 3.15-µg dose and full-length spike vaccine at 15-µg dose. To check whether the low neutralizing titre of T2_17_TM was due to the low dose of 3.15 µg, we tested the sera of guinea pigs immunized with 15 µg of T2_17_TM and compared them with sera of guinea pigs immunized with 15 µg of full-length spike. The sera from guinea pigs immunized with 15 µg of T2_17_TM neutralized pseudoviruses expressing the XBB.1.5 variant (Fig. 5d).

## Discussion

Two human SARS epidemics were caused in the past two decades by zoonotic sarbecoviruses that infect through the ACE-2 receptor. Vaccines that can provide broad protection against such lineages are urgently needed. Strategies to achieve pan-sarbecovirus and pan-betacoronavirus protection include synthetic mRNAs expressing chimaeric versions of different spike proteins^29^, and mosaic or cocktail nanoparticles expressing RBDs derived from different coronaviruses^30^. These strategies have been reported to be effective in generating immune responses to pan-sarbecoviruses and pan-betacoronaviruses but require the synthesis, manufacture and formulation of multiple gene constructs, which adds regulatory and technical complexity to large-scale manufacturing. In addition to the zoonotic spillover from the related bat or other mammal sarbecoviruses, another cause of concern is the rapid accumulation of immune-escape mutations in circulating SARS-CoV-2. Since late 2020, many mutations leading to immune escape or to increased transmissibility (or to both) have been reported, with the latest circulating Omicron lineage reporting the greatest number of mutations in the SARS-CoV-2 viral genome^14^. An ideal vaccine candidate targeting circulating and emerging VOCs would be a single antigen providing protection against the diverse group of sarbecoviruses.

In this study we report preclinical data for a single-antigen RBD-subunit-based vaccine that induces immune responses against SARS-CoV, SARS-CoV-2, RaTG13, WIV16 and VOCs. The core backbone of the antigen was designed using DIOSynVax technology. It integrates phylogenetic relationships between the input sequences and structural bioinformatics to generate a core antigen sequence that ideally should generate immune responses against a diverse group of phylogenetically related viruses. We further modified the core antigen sequence by mutating some of the known epitopes within the RBD or by introducing a glycosylation site (or both) to enhance the immunogenicity of the antigen. This resulted in a panel of antigens, referred to as T2_13 to T2_17. The designs were generated in June 2020 and the epitope information was derived from the structural data available in June 2020. Epitope regions of CR3022 (ref. ^16^), S309 (ref. ^15^) and B38 (ref. ^11^) were considered for these designs. Antibodies binding to the RBD were later classified into four classes (classes 1–4)^31^. Out of the four classes, three classes (namely, class 1, class 2 and class 3) are potent neutralizers. The antibody B38 belongs to class 1, S309 to class 3 and CR3022 to class 4. By introducing a glycosylation site at an epitope of B38, we masked the epitope region corresponding to class-1 antibodies, while the antibodies targeting class 2 and class 3 are still available in the constructs and would induce neutralizing antibodies. The introduction of glycosylation was aimed to mask one of the divergent epitopes and to re-rank the order of the epitope presentation to the immune system, thus generating a pool of polyclonal sera enriched in both neutralizing and variant-resilient antibodies.

The strength and breadth of immune responses against different spike proteins were confirmed in BALB/c mice. From the binding profile of the sera of mice immunized with the designed antigens, we selected T2_17 for further preclinical studies. Mice immunized with T2_17 as a DNA immunogen produced significant binding antibody titres against both SARS-CoV and SARS-CoV-2. Neutralizing antibodies were also measured in outbred guinea pigs and outbred rabbits against both SARS-CoV and SARS-CoV-2. Notably, rabbit sera neutralized a wide panel of SARS-CoV-2 VOCs, namely Alpha, Beta, Gamma, Delta and Omicron BA.1. These broad humoral responses validate the use of the DIOSynVax technology for the generation of a pan-sarbecovirus RBD-based antigen. The breadth of the antibody immune responses elicited by the antigen T2_17 against VOCs up to BA.1 is particularly encouraging, as the antigen was designed using the hCoV-19/Wuhan/IVDC-HB-01/2019 strain of SARS-CoV-2, suggesting the applicability of the technology to capture some of the future variants, if not all of them.

To assess the suitability of a T2_17 antigen as a booster within the background of the non-naive population, K18-hACE-2 mice were primed with the AZD1222 vaccine and boosted with AZD1222 or T2_17 as a DNA or MVA-vectored immunogen at 4-week intervals and challenged with either the Victoria or Delta strains of SARS-CoV-2. All the antigen-immunized mice were protected against the challenge, with increases in neutralizing antibody titres (4 weeks after boost) against Delta in the T2_17-boosted group. Neutralizing antibodies against SARS-CoV were observed in the T2_17(MVA) group. T2_17(DNA) did not induce neutralizing antibodies against SARS-CoV. We believe that this is due to the difference in vaccine vector between AZD1222 and DNA, but this has not been addressed in the present study. Responses against SARS1 were substantially better in the group that was boosted with T2_17(MVA). A longitudinal serology study was carried out to understand the influence of boosting K18-hACE-2 mice at 20 weeks post prime. The antibody titres remained high for 12 weeks post prime. Four weeks after boosting, the T2_17(MVA)-boosted group showed higher neutralizing antibody titres than other groups. Equivalent neutralizing antibody titres were observed for all the boosted groups at terminal bleed. Peptide microarrays were used on the terminal sera of the K18-hACE-2 mice from the longitudinal study to check for differential immune responses in different boost groups. A higher number of peptide hits for the RBD regions were observed for the T2_17(MVA)-boosted group, suggesting a greater elicitation of the RBD-specific humoral response in the regime of adenovirus prime and MVA boost.

Considering the important successes of mRNA vaccines in the current COVID-19 pandemic^32^, T2_17 was tested as an mRNA immunogen in mice and guinea pigs using chemically modified mRNA^17^ in an LNP formulation^18^. A previous report on an mRNA vaccine has shown that a membrane-anchored and prefusion-stabilized full-length MERS spike antigen elicited more potent pseudovirus-neutralizing antibody responses than the soluble form^28^. In our study, in addition, T2_17 was also delivered as a transmembrane-anchored form (T2_17_TM) mRNA immunogen to mice at different doses of 5 µg and 10 µg. T2_17_TM showed significantly higher binding antibody titres than T2_17 at the lower dose of 5 µg, but both had comparable binding antibody titres at the 10 µg dose. On the basis of these observations, T2_17_TM was further validated as an mRNA immunogen in guinea pigs. Both binding and neutralizing antibodies were induced by T2_17_TM. Six weeks post boost, only guinea pigs immunized with T2_17_TM showed neutralizing antibodies against SARS-CoV and the SARS-CoV-2 Omicron (BA.1) variant at the lower dose of 3.15 µg and against XBB.1.5 at the higher dose of 15 µg. The group immunized with the full-length SARS-CoV-2 spike did not show a robust neutralizing immune response against SARS-CoV or the SARS-CoV-2 Omicron variants at the 15-µg dose. Higher antibody titres against SARS-CoV-2 and RaTG13 were observed for the full-length spike, but it must be noted that the full-length homotrimer spike presents three RBD subunits that are homologous to the SARS-CoV-2 spike tested here and would always induce higher titres in comparison with any other heterologous antigens. Moreover, the high similarity between the S2 regions of SARS-CoV-2 and RaTG13 would also induce higher cross-neutralizing antibodies between RaTG13 and SARS-CoV-2 and would lead to higher antibody titres for the full-length spike in comparison with T2_17.

At the time of the design of T2_17, none of the SARS-CoV-2 variants had yet been observed. Although T2_17 still generates neutralizing antibodies against VOCs including the recent XBB.1.5, the titres are lower than observed against the Wuhan strain. With the extraordinary variation owing to the global distribution of SARS-CoV-2 in animals and humans, future updates may be needed for T2_17, such as including the sequence information of the VOCs as well as combining it with other conserved structural and non-structural antigens. Furthermore, to understand the immunogenicity of T2_17 in the background of the current complex immunity observed in the human population, phase-1 clinical trials have now been initiated.

All the studies combined indicate that T2_17 is an efficacious single antigen for targeting multiple sarbecoviruses and support its applicability across different vaccine technologies. Immunization with T2_17 generated a robust humoral immune response against SARS-CoV, SARS-CoV-2, RaTG13, WIV16 and the SARS-CoV-2 variants Alpha, Beta, Gamma, Delta and Omicron (BA.1, XBB.1.5). That the design predated the emergence of these VOCs and none of the sequences were included in the initial design is strong validation of the efficacy of the DIOSynVax technology. Given the continuous emergence of new variants, new vaccine antigens should be substantially different from the Wuhan strain or from other variants to surpass the boosting of the immunodominant epitopes conserved in these strains^33,34^. All the current vaccines use the full-length spike as the antigen and only 16% of the antibodies generated against the spike antigen are RBD-directed^35^. As T2_17 is an RBD-based antigen substantially differing from the Wuhan-Hu-1 strain of SARS-CoV-2, it could be used as a booster vaccine candidate for overcoming immune imprinting by vaccines that use the full-length spike.

## Methods

### Phylogenetic analysis

Protein sequences of spike proteins were downloaded from the NCBI virus database for all the known sarbecoviruses (June 2020). A multiple sequence alignment was generated using MUSCLE^36^. The resulting multiple sequence alignment was pruned to the RBD region, filtered at 95% sequence identity and used as input for phylogenetic tree reconstruction. The phylogenetic tree was generated with IQ-TREE^37^ using the protein model with the best Bayesian information criterion score. The resultant tree was used for generating the phylogenetically optimized design using HyPhy^38^.

### Epitope identification

Available structural data (June 2020) for spike protein–antibody complexes for SARS-CoV and SARS-CoV-2 were downloaded from the Protein Data Bank (PDB)^39^. Structural data were then pruned for antigen–antibody complexes where the epitopes were on the RBD. Amino acid residues of antigen that have at least one atom within 5 Å radii of at least one atom of an amino acid of the antibody were defined as epitope residues, with epitope regions defined as contiguous stretches of at least 5 amino acids.

### Glycosylation site modification

The position of the glycosylation site was determined by in silico mutation of triplets of amino acids in the epitopes to the glycosylation sequon, N-X-T/S^40^, using the FoldX algorithm^24^. Briefly, residues succeeding the N-X motif, where X can be any amino acid except proline, were mutated to either threonine or serine, or residues preceding X-T/S, where X can be any amino acid except proline, were mutated to asparagine to generate N-X-T/S motifs. The mutations with the least energy cost as calculated by the Build module of FoldX^24^ were selected.

### Molecular modelling

Structural models were generated for T2_13 using MODELLER^41,42^, with both SARS-CoV and SARS-CoV-2 structures as templates. The structural model with the highest discrete optimized potential energy score^43^ was chosen as the working model for further molecular modelling. The side chains for the model were further optimized using SCWRL^44^ and energy minimized using GROMACS^45^. For T2_14 to T2_18, mutations were introduced using T2_13 as the reference structure, utilizing the BUILD module of the FoldX algorithm^24^, and checked for structural stability using the FoldX forcefield^24^.

### Production and transformation of plasmids

Sequences of antigens were codon optimized for expression in humans via the GeneOptimizer algorithm^46^. These genes were cloned into pEVAC plasmid (GeneArt) via restriction digestion. Plasmids were transformed via heat shock in chemically induced competent *E. coli* DH5α cells (Invitrogen, 18265-017). Plasmid DNA was extracted from transformed bacterial cultures via the Plasmid Mini Kit (Qiagen, 12125). All plasmids were subsequently quantified using UV spectrophotometry (NanoDrop, Thermo Scientific)^46^.

### Vaccination experiments in mice

Ten groups of six 8–10-week-old female BALB/c mice were purchased from Charles River Laboratories. Mice were immunized a total of 4 times at 30-d intervals. A total volume of 50 µl of PBS containing 50 µg of plasmid DNA was administered via subcutaneous route in the rear flank. Blood was sampled from the saphenous vein at 15-d intervals and animals were terminally bled by cardiac puncture under non-recovery anaesthesia at day 150.

### Fluorescence-activated cell sorting (FACS) assay

HEK293T cells were transfected with an expression plasmid expressing wild-type spike glycoprotein of each of the four ACE-2 binding sarbecoviruses including SARS-CoV (SARS-Tor2), SARS-CoV-2 (hCoV-19/Wuhan/IVDC-HB-01/2019), WIV16 (accession ID: ALK02457) and RaTG13 (accession ID: QHR63300). At 48 h after transfection, cells were transferred into V-bottom 96-well plates (50,000 cells per well). Cells were incubated with sera (diluted at 1:50 in PBS) or anti-mouse IgG isotype negative control (Invitrogen, 10400C, diluted to 20 µg ml^−1^ in PBS) for 30 min, washed with FACS buffer (PBS, 1% FBS, 0.02% Tween 20) and stained with goat anti-mouse IgG (H + L) Alexa Fluor 647 secondary antibody (Invitrogen, A32728, diluted at 20 µg ml^−1^ in FACS buffer) for 30 min in the dark. Cells were washed with FACS buffer and samples were run on an Attune NxT flow cytometer (Invitrogen) with a high-throughput auto sampler. Dead cells were excluded from the analysis by staining cells with 7-aminoactinomycin D (7-AAD) and gating 7-AAD-negative live cells (Supplementary Fig. 3).

### ELISA

The assays were adapted from those originally described in ref. ^47^. Briefly, Nunc MaxiSorp flat-bottom plates were coated with 50 μl per well of 1 μg ml^−1^ RBD from SARS-CoV (SARS1) or SARS-CoV-2 (SARS2) in DPBS (−Ca^2+^/−Mg^2+^) and incubated overnight at 4 °C. The next day, the plates were blocked with 3% milk in PBST (0.1% w/v Tween 20 in PBS) for 1 h. After removing the blocking buffer, 50 μl per well of serum samples diluted in PBST-NFM (1% w/w non-fat milk in PBST) was added to the plates and incubated on a plate shaker for 2 h at 20 °C. The plates were washed 3 times with 200 μl of PBST, and then 50 μl of HRP-conjugated goat anti-mouse IgG (H and L chains) (Jackson ImmunoResearch) was added to each well and left to incubate on a plate shaker for 1 h. Plates were washed 3 times with 200 μl of PBST before 50 μl per well of 1-Step Ultra TMB chromogenic substrate (Sigma) was added to the plates and the chemical reaction was stopped 3 min later with 50 μl 2 N H_2_SO_4_. The optical density at a wavelength of 450 nm (OD_450_) was measured using a BioRad microplate reader. Values from the dilution curve were used to determine the area under the curve.

### Intradermal nucleic acid immunization in guinea pigs

Two groups of eight 7-week-old female Dunkin Hartley guinea pigs (Envigo RMS) were immunized a total of 3 times at 28-d intervals. A total volume of 200 µl of PBS containing 400 µg of plasmid DNA was administered using the PharmaJet Tropis intradermal device, split over each hind leg. Blood was sampled from the saphenous vein at 14-d intervals.

### Intradermal nucleic acid immunization in rabbits

Ten mature (5 male, 5 female) rabbits were immunized with a good manufacturing practice lot of pEVAC_T2_17 (clinical pEVAC_PS) intradermally using PharmaJet Tropis needleless delivery to the upper left and right hind limbs (300 µl at 2 mg ml^−1^). For the control group, 10 mature (5 male, 5 female) rabbits were injected with PBS. Arterial blood was sampled at 14-d intervals.

### Production of lentiviral pseudotypes

Lentiviral pseudotypes were produced by transient transfection of HEK293T/17 cells with packaging plasmids p8.91 (refs. ^48,49^) and pCSFLW^50^ and different SARS-CoV-2 VOC spike-bearing expression plasmids using the FuGENE HD Transfection Reagent^51,52^. Supernatants were collected after 48 h, passed through a 0.45 µm cellulose acetate filter and titrated on HEK293T/17 cells transiently expressing human ACE-2 and TMPRSS2. Target HEK293T/17 cells were transfected 24 h earlier with 2 µg pCAGGS-huACE-2 and 75 ng pCAGGS-TMPRSS2 (refs. ^53,54^).

### Pseudotype-based microneutralization assay

Pseudotype-based microneutralization assays (pMN) were performed as described previously^55^. Briefly, serial dilutions of serum were incubated with SARS-CoV-2, RaTG13, SARS-CoV, WIV16 and SARS-CoV-2 variant spike bearing lentiviral pseudotypes for 1 h at 37 °C and 5% CO_2_ in 96-well white cell culture plates. HEK293T/17 cells (1.5 × 10^4^) transiently expressing human ACE-2 and TMPRSS2 were then added per well and plates incubated for 48 h at 37 °C and 5% CO_2_ in a humidified incubator. Bright-Glo (Promega) was then added to each well and luminescence read after a 5-min incubation period. Experimental data points were normalized to 100% and 0% neutralization controls, and nonlinear regression analysis performed in GraphPad Prism 9 to produce neutralization curves and half-maximal inhibitory concentration (IC_50_) values.

### ACE-2 competition assay

The SARS-CoV-2 surrogate virus neutralization test (SVNT, Genscript) was carried out following manufacturer instructions. Briefly, sera were diluted in PBS across an 8 point 1:2 dilution series from a starting concentration of 1:50. Samples were further diluted in the provided sample buffer at a 1:9 ratio and then mixed with HRP conjugated to SARS-CoV-2 RBD protein, incubated at 37 °C for 30 min and added to human ACE-2 protein coated wells in 96-well plate format. The reaction was incubated at 37 °C for 15 min and then washed 4 times with the provided wash buffer. TMB solution was then added and the plates were incubated for 15 min in the dark at room temperature to allow the reaction to develop. The reaction was then quenched using the provided stop solution and absorbance was read at 450 nm.

### MVA production

The MVA strain used in this study was MVA-CR19. Recombinant MVA that expresses SARS-CoV-2 RBD T2-17 was generated as described previously^56^. In brief, for in vivo recombination, adherent AGE1.CR.pIX were infected with parental MVA-CR19 TK-GFP at different multiplicity of infection (MOIs) ranging from 0.5 to 0.006 particle forming unit. After 2 h, the cells were transfected with 0.4 µg of the shuttle vector pMVA_RBD T2_17 using Effectene (Qiagen) according to manufacturer instructions. After 48 h, the cells were collected, lysed using three freeze–thaw cycles and sonicated. Pure recombinant viruses were obtained by sequential plaque purification under agarose overlays and confirmed to be free of contaminating parental MVA-CR19 TK-GFP by PCR screening. This recombinant MVA encoding SARS-CoV-2 RBD T2-17 was plaque purified for an additional three rounds. The resulting recombinant MVA-CR19 RBD-T2_17 (MVA T2_17) virus stock was produced in suspension AGE1.CR.pIX cells, purified via two ultracentrifugation rounds over a 35% sucrose cushion and titrated on DF-1 cells using crystal violet staining. The sequence of recombinant MVA and the absence of revertant MVA was confirmed by PCR amplification and Sanger sequencing. The expression of RBD T2_17 was confirmed by western blot analysis with monoclonal antibody CR3022, with cell lysates from HEK293 cells collected 24 h after infection (MOI 2) with MVA T2_17.

### Vaccine boost efficacy studies in K18-hACE-2 mice

Eight groups of six 8–15-week-old homozygous female K18-hACE-2 mice (Jax) were primed with 1.4 × 10^9^ viral particles of AZD1222 or PBS by intramuscular route, in a total volume of 100 µl split over the two rear legs. After 28 d, 2 groups of 6 mice were boosted with either PBS, AZD1222, T2_17(DNA) or T2_17(MVA). Mice were bled at 2-week intervals and challenged at day 84 with either Victoria/1/2020 (B-lineage) or Delta SARS-CoV-2 by intranasal route, in a total volume of 40 µl over both nares. Mice were weighed daily and monitored for clinical signs for a period of 14 d before being humanely culled by terminal bleed.

### Longitudinal serology studies in K18-hACE-2 mice

Six groups of six 8–15-week-old homozygous female K18-hACE-2 mice (Jax) were primed with 1.4 × 10^9^ viral particles of AZD1222 or PBS by intramuscular route, in a total volume of 100 µl split over the two rear legs. After 20 weeks, groups of 6 mice were boosted either with PBS, AZD1222, T2_17(DNA) or T2_17(MVA). Mice were bled at 12 and 24 weeks post prime, and terminally bled at week 44 post prime.

### Peptide microarray

Four samples were selected from the terminal bleeds of the K18-hACE-2 mice used for the longitudinal study (ACE-2AZD1222 vaccine prime, T2_17 boost samples). The samples were selected on the basis of the quality of serum and performance in the pMN assay against SARS-CoV-2 Wuhan. Aliquots (30 µl) of each sample were sent to PEPperPRINT for peptide microarray analysis. Briefly, 15-mer peptides spanning the SARS-CoV RBD (213 amino acid (aa)), SARS-CoV-2 RBD (214 aa) and T2_17 RBD (214 aa) with a 14-aa overlap were printed in duplicate per array copy for a total of five array copies. HA and c-Myc control peptides were included in each array copy. The protein sequences were elongated by neutral GSGSGSG linkers to avoid truncated peptides, and identical peptides were removed. In total, 1,310 peptide sequences were synthesized and spotted in duplicate onto the PEPperCHIP microarray platform. The corrected raw intensities were log transformed for all the serum samples. For each vaccine group, namely PBS/T2_17MVA, AZD1222/AZD1222 and AZD1222/T2_17MVA, the peptides with raw intensities twofold higher than the maximum intensity observed in the PBS/PBS group were considered as antibody epitope hits.

### mRNA vaccine production

mRNA sequences encoding the SARS-CoV-2 spike protein with 2 proline mutations, T2_17 and T2_17_TM, were synthesized by in vitro transcription from linearized plasmid DNA templates using modified nucleotides to generate partial modified mRNAs. After in vitro transcription, mRNAs were dephosphorylated and enzymatically polyadenylated. Purification steps were performed by precipitation and mRNA subsequently formulated in water for injection at a concentration of 1 mg ml^−1^. mRNAs were stored at −80 °C until LNP encapsulation. Each mRNA was LNP encapsulated via nanoprecipitation by microfluidic mixing of mRNA in citrate buffer (pH 4.5) with ionizable, structural, helper and polyethylene glycol lipids in ethanol, followed by buffer exchange and concentration via tangential flow filtration. mRNA LNPs were filtered through a 0.2-μm membrane and stored at −20 °C until use. The drug product was analytically characterized and evaluated as acceptable for in vivo use.

### Immunization of BALB/c mice with mRNA

Seven groups of six 8–10-week-old female BALB/c mice were purchased from Charles River Laboratories. Mice were immunized twice at a 21-d interval. A total volume of 100 µl of vehicle containing various amounts of mRNA was administered via intramuscular route in each hind leg. Blood was sampled from the saphenous vein at 21-d intervals and animals were terminally bled by cardiac puncture under non-recovery anaesthesia at day 63.

### Immunization of guinea pigs with mRNA

Three groups of six 8–10-week-old female Hartley guinea pigs were purchased from Envigo. Guinea pigs were immunized twice at a 21-d interval. A total volume of 200 µl of vehicle containing various amounts of mRNA (3.15 µg or 15 µg) was administered via intramuscular route in each hind leg. Blood was sampled from the saphenous vein at 21-d intervals and animals were humanely euthanized after the final bleed on day 63.

### Statistical analyses

Two-tailed Mann–Whitney *U* tests were performed for all the comparisons using the Python sklearn package^57^. All plots were generated using the Python Matplotlib package and statannotat package^58^.

### Animal-work ethics

Animal studies were approved by the Animal Welfare and Ethical Review Body, University of Cambridge, and experiments were carried out under an approved UK home office licence (P8143424B).

### Reporting summary

Further information on research design is available in the Nature Portfolio Reporting Summary linked to this article.

## Supplementary information

Supplementary Information Supplementary Figs. 1–3.

Reporting Summary

## Data Availability

The main data supporting the results in this study are available within the paper and its Supplementary Information. The sequences used for designing the vaccine antigens were retrieved from the publicly available NCBI virus database. The structure coordinates of the antigen–antibody complexes used for the analyses are available in the Protein Data Bank. The sequences of the antigens have been patented under UK Patent Application No. 2303150.3, Coronavirus Vaccines.
