# Supplementary material for: A computationally designed antigen eliciting broad humoral responses against SARS-CoV-2 and related sarbecoviruses
Source: Nat Biomed Eng. 2023 Sep 25;9(2):153–66. doi: 10.1038/s41551-023-01094-2 (PMC11839467; doi:10.1038/s41551-023-01094-2)
Supplement: Supplementary file 1 — Supplementary Figs. 1–3. [file 41551_2023_1094_MOESM1_ESM.pdf]

nature biomedical engineering

Article https://doi.org/10.1038/s41551-023-01094-2

A computationally designed antigen eliciting broad humoral responses against SARS-CoV-2 and related sarbecoviruses

In the format provided by the authors and unedited

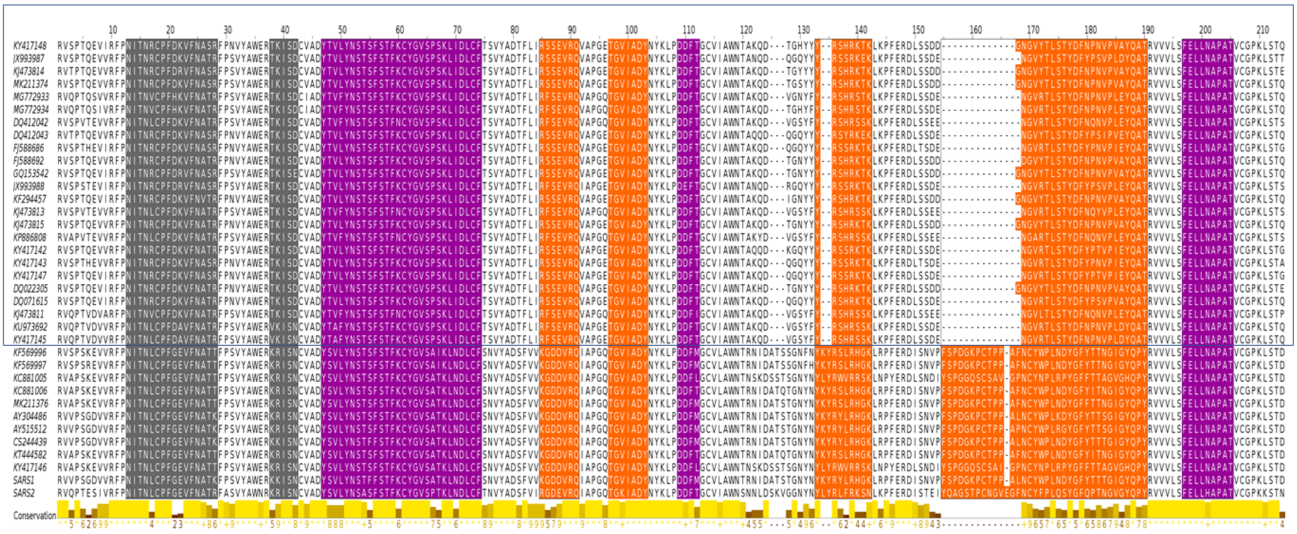

Supplementary Fig. 1 | Multiple sequence alignment of the RBD of known sarbecoviruses. Sarbecoviruses are divided into two distinct phylogenetic clades – clade 1 (boxed in blue) and clade 2. Members of clade 1 have deletions within the ACE-2 binding motif and have been reported to not bind human ACE-2 receptor. The epitopes targeted by the S309, CR3022, and B38 antibodies are coloured in grey, purple, and orange, respectively.

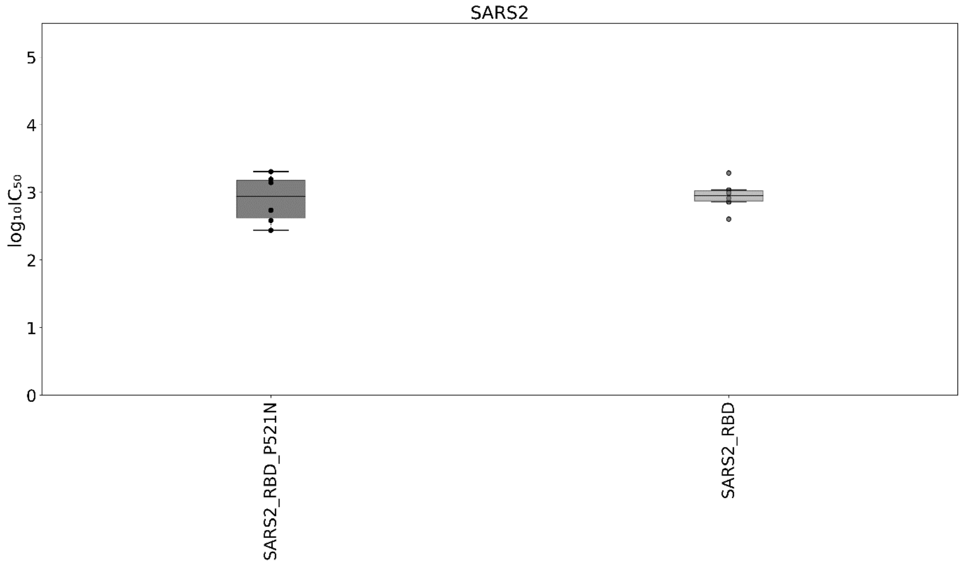

Supplementary Fig. 2 | Neutralisation data for SARS2_RBD_P521N and SARS2_RBD in BALB/c mice. Sera from BALB/c mice immunised with SARS2_RBD_P521N and SARS-COV-2 RBD generated similar neutralising antibody responses 14 days after the fourth immunisation. The X-axis represents the antigens, and the Y-axis represents the $\log_{10}IC_{50}$ values for neutralisation curves. The difference is statistically non-significant (Two-tailed Mann-Whitney U test, p-value = 0.4681). The boxes represent the quartiles (25th, 50th and 75th percentiles) of the distribution, and the whiskers represent the minimum and maximum of the distribution (excluding outliers) and the fliers represented as filled circle represent the outliers.

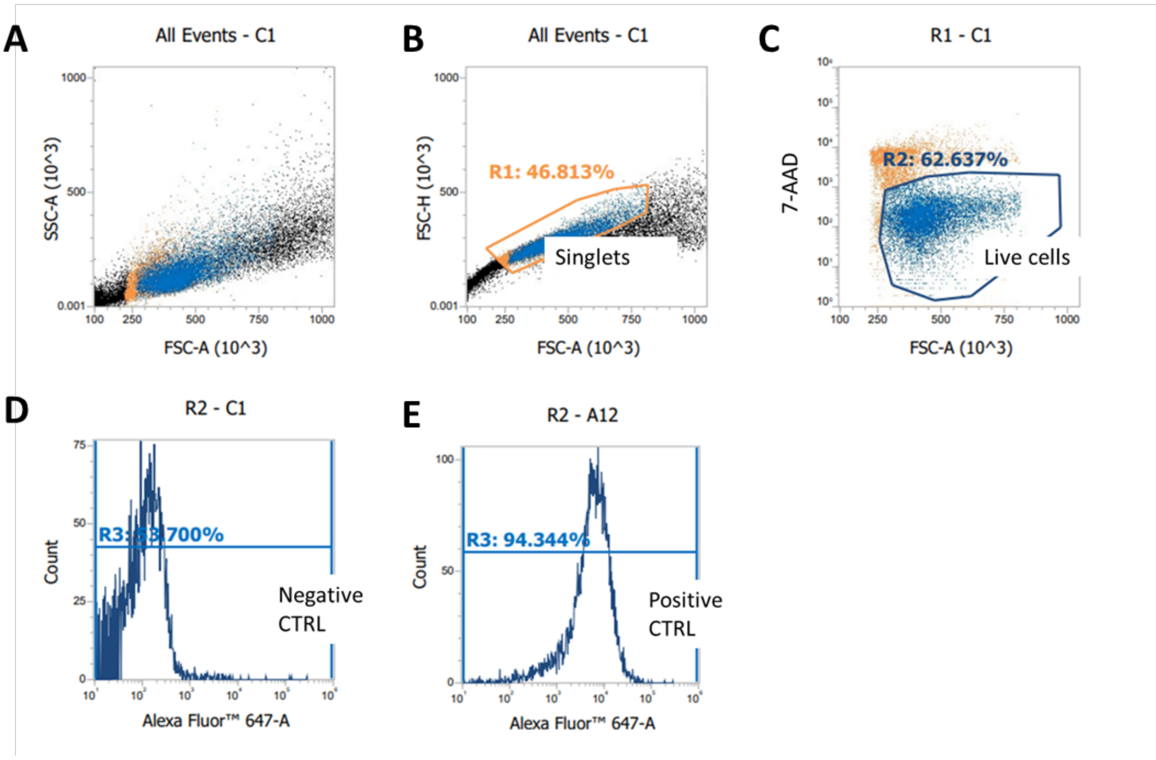

Supplementary Fig. 3 l Gating strategy used to analyze flow cytometry data.
(**A**) Preliminary FSC/SSC gates were set on the starting cell population. (**B**) Singlets were gated by plotting FSC-H versus FSC-A, followed by gating live cells as 7-AAD negative (**C**). (**D**) Live cells were visualized as a histogram in the RL-1 channel, with the PMT of the negative cell population set between 10^2 and 10^3. (**E**) The MFI values of positive control is shown. Representative flow cytometry plots are shown.
