# Supplementary material for: A computationally designed antigen eliciting broad humoral responses against SARS-CoV-2 and related sarbecoviruses
Source: Nat Biomed Eng. 2023 Sep 25;9(2):153–66. doi: 10.1038/s41551-023-01094-2 (PMC11839467; doi:10.1038/s41551-023-01094-2)
Supplement: Supplementary file 2 — Reporting Summary [file 41551_2023_1094_MOESM2_ESM.pdf]

nature portfolio

Corresponding author(s): Jonathan Heeney
Last updated by author(s): Aug 18, 2023

Reporting Summary

Nature Portfolio wishes to improve the reproducibility of the work that we publish. This form provides structure for consistency and transparency in reporting. For further information on Nature Portfolio policies, see our Editorial Policies and the Editorial Policy Checklist.

Statistics

For all statistical analyses, confirm that the following items are present in the figure legend, table legend, main text, or Methods section.

n/a	Confirmed	
☐	☒	The exact sample size (*n*) for each experimental group/condition, given as a discrete number and unit of measurement
☐	☒	A statement on whether measurements were taken from distinct samples or whether the same sample was measured repeatedly
☐	☒	The statistical test(s) used AND whether they are one- or two-sided<br>*Only common tests should be described solely by name; describe more complex techniques in the Methods section.*
☒	☐	A description of all covariates tested
☒	☐	A description of any assumptions or corrections, such as tests of normality and adjustment for multiple comparisons
☐	☒	A full description of the statistical parameters including central tendency (e.g. means) or other basic estimates (e.g. regression coefficient) AND variation (e.g. standard deviation) or associated estimates of uncertainty (e.g. confidence intervals)
☐	☒	For null hypothesis testing, the test statistic (e.g. *F*, *t*, *r*) with confidence intervals, effect sizes, degrees of freedom and *P* value noted<br>*Give P values as exact values whenever suitable.*
☒	☐	For Bayesian analysis, information on the choice of priors and Markov chain Monte Carlo settings
☒	☐	For hierarchical and complex designs, identification of the appropriate level for tests and full reporting of outcomes
☒	☐	Estimates of effect sizes (e.g. Cohen's *d*, Pearson's *r*), indicating how they were calculated

Our web collection on statistics for biologists contains articles on many of the points above.

Software and code

Policy information about availability of computer code

Data collection	Sequence data were downloaded from the NCBI virus database using the NCBI download tool.<br>Structure data were downloaded from the PDB using the PDB download tool.
Data analysis	Sequence cleaning and pruning were done using custom codes written in python 3.<br>Sequence alignments were generated using the MUSCLE algorithm version 3.8.<br>The phylogenetic tree was built using the iQtree algorithm version 1.6.1 with the in-built statistical packages.<br>Novel sequences were designed using the HyPhy package, version 2.5.<br>Structure visualization and the rendering of images were done using PyMol ver 2.5.<br>Stability calculations were done using the FOLDX 5 algorithm.<br>Energy minimization was done using the Gromacs-2019.4 package with in-built force-fields and parameters.<br>Plots were generated using custom codes written in python and using the matplotlib package.<br>Statistical analyses were done using the numpy package in python 3.<br>All the software was used with Academic license.

For manuscripts utilizing custom algorithms or software that are central to the research but not yet described in published literature, software must be made available to editors and reviewers. We strongly encourage code deposition in a community repository (e.g. GitHub). See the Nature Portfolio guidelines for submitting code & software for further information.

Data

Policy information about availability of data

All manuscripts must include a data availability statement. This statement should provide the following information, where applicable:

- Accession codes, unique identifiers, or web links for publicly available datasets
- A description of any restrictions on data availability
- For clinical datasets or third party data, please ensure that the statement adheres to our policy

The main data supporting the results in this study are available within the paper and its Supplementary Information. The sequences used for designing the vaccine antigens were retrieved from the publicly available NCBI virus database. The structure coordinates of the antigen–antibody complexes used for the analyses are available in the Protein Data Bank. The sequences of the antigens have been patented under UK Patent Application No. 2303150.3, Coronavirus Vaccines.

Research involving human participants, their data, or biological material

Policy information about studies with human participants or human data. See also policy information about sex, gender (identity/presentation), and sexual orientation and race, ethnicity and racism.

Reporting on sex and gender	The study did not involve human participants.
Reporting on race, ethnicity, or other socially relevant groupings	—
Population characteristics	—
Recruitment	—
Ethics oversight	—

Note that full information on the approval of the study protocol must also be provided in the manuscript.

Field-specific reporting

Please select the one below that is the best fit for your research. If you are not sure, read the appropriate sections before making your selection.

☒ Life sciences ☐ Behavioural & social sciences ☐ Ecological, evolutionary & environmental sciences

For a reference copy of the document with all sections, see nature.com/documents/nr-reporting-summary-flat.pdf

Life sciences study design

All studies must disclose on these points even when the disclosure is negative.

Sample size	We used groups of 6 mice as the lowest number of mice suitable for performing the statistical analyses.
Data exclusions	No data were excluded from the analyses.
Replication	All the reported experiments were done in duplicate.
Randomization	The animals in all the animals studies were randomly assigned to groups.
Blinding	The investigators were blinded to the assigned groups.

Reporting for specific materials, systems and methods

We require information from authors about some types of materials, experimental systems and methods used in many studies. Here, indicate whether each material, system or method listed is relevant to your study. If you are not sure if a list item applies to your research, read the appropriate section before selecting a response.

Materials & experimental systems

n/a	Involved in the study	
☐	☒	Antibodies
☐	☒	Eukaryotic cell lines
☒	☐	Palaeontology and archaeology
☐	☒	Animals and other organisms
☒	☐	Clinical data
☒	☐	Dual use research of concern
☒	☐	Plants

Methods

n/a	Involved in the study	
☒	☐	ChIP-seq
☐	☒	Flow cytometry
☒	☐	MRI-based neuroimaging

Antibodies

Antibodies used	Clone CR3022 (absolute antibody Ab01680-10.0 and Ab01680-3.0).<br>IgG Isotype negative control (Invitrogen 10400C).<br>Alexa Fluor 647 Secondary Antibody (Invitrogen A32728).<br>HRP-conjugated goat anti Ig (H and L chains) (Jackson ImmunoResearch, 109-035-088 (anti-human), and 706-035-148 (anti-guinea pig)).<br>HRP-conjugated goat anti Ig (H and L chain) (Thermo Fischer Scientific, 31460 (anti-rabbit)).
Validation	All antibodies used were from commercial sources and validated by them.

Eukaryotic cell lines

Policy information about cell lines and Sex and Gender in Research

Cell line source(s)	HEK293T/17, from ATCC.<br>HEK293T, from ATCC.<br>Suspension of AGE1.CR.pIX cells, from ProBioGenAG.
Authentication	None of the cell lines used were authenticated.
Mycoplasma contamination	All cells lines were tested, and confirmed negative for mycoplasma.
Commonly misidentified lines<br>(See ICLAC register)	No commonly misidentified cell lines were used.

Animals and other research organisms

Policy information about studies involving animals; ARRIVE guidelines recommended for reporting animal research, and Sex and Gender in Research

Laboratory animals	BALB/c mice (BALB/cAnNCrl Charles River), 8-to-10-weeks old, female (Charles River Laboratories).<br>Homozygous K18-hACE2 mice (B6.Cg-Tg(K18-ACE2)2Prlmn/J, Jax), 8-to-15-weeks old, female (Charles River Laboratories).<br>All mice used in this study were female, 8–15 weeks old and weighing 20–30g at the time of use.<br>Dunkin Hartley Guinea pigs, 7-week-old female (Envigo).<br>Rabbits, 5 female and 5 male (Charles River Laboratories).
Wild animals	The study did not involve wild animals.
Reporting on sex	Only females were used in this study, with the exception of rabbits (50% male, 50% female).
Field-collected samples	The study did not involve samples collected from the field.
Ethics oversight	UK Home Office License P8143424B. Characterization of vaccine candidates against viral diseases. Each individual animal study was submitted to a portal to the local AWERB University of Cambridge.

Note that full information on the approval of the study protocol must also be provided in the manuscript.

Flow Cytometry

Plots

Confirm that:

☒ The axis labels state the marker and fluorochrome used (e.g. CD4-FITC).

☒ The axis scales are clearly visible. Include numbers along axes only for bottom left plot of group (a 'group' is an analysis of identical markers).

☒ All plots are contour plots with outliers or pseudocolor plots.

☒ A numerical value for number of cells or percentage (with statistics) is provided.

Methodology

Sample preparation	Transfected HEK293T (from ATCC).
Instrument	Attune NxT Flow Cytometer
Software	Attune Cytometric Software
Cell population abundance	Not applicable for binding studies.
Gating strategy	Preliminary FSC/SSC gates were set on the starting cell population. Singlets were then gated by plotting FSC-H versus FSC-A, followed by gating live cells as 7-AAD negative. The live-cell population was visualized as a histogram in the RL-1 channel. The PMT of the negative cell population was set between 10^2 and 10^3 to reduce background noise. The MFI values of the negative population, which consisted of untransfected cells stained with primary and secondary AF647 antibodies, were subtracted from the MFI of the test samples to correct for non-specific binding.

☒ Tick this box to confirm that a figure exemplifying the gating strategy is provided in the Supplementary Information.
